# Risk of pneumonia among residents living near goat and poultry farms during 2014-2016

**DOI:** 10.1371/journal.pone.0223601

**Published:** 2019-10-14

**Authors:** Pim M. Post, Lenny Hogerwerf, Anke Huss, Ronald Petie, Gert Jan Boender, Christos Baliatsas, Erik Lebret, Dick Heederik, Thomas J. Hagenaars, C. Joris IJzermans, Lidwien A. M. Smit

**Affiliations:** 1 National Institute for Public Health and the Environment, Bilthoven, the Netherlands; 2 Institute for Risk Assessment Sciences, Utrecht University, Utrecht, the Netherlands; 3 Netherlands Institute for Health Services Research, Utrecht, the Netherlands; 4 Wageningen Bioveterinary Research, Lelystad, the Netherlands; The University of Hong Kong, CHINA

## Abstract

In the Netherlands, an association was found between the prevalence of pneumonia and living near goat and poultry farms in 2007–2013. This association then led to regulatory decisions to restrict the building of new goat farms and to reduce emissions of poultry farms. Confirmation of these results, however, is required because the period of previous analyses overlapped a Q-fever epidemic in 2007–2010. To confirm the association, we performed a population-based study during 2014–2016 based on general practitioner (GP) data. Electronic medical records of 90,183 persons were used to analyze the association between pneumonia and the population living in the proximity (within 500–2000 m distance) of goat and poultry farms. Data were analyzed with three types of logistic regression (with and without GP practice as a random intercept and with stratified analyses per GP practice) and a kernel model to discern the influence of different statistical methods on the outcomes. In all regression analyses involving adults, a statistically significant association between pneumonia and residence within 500 meters of goat farms was found (odds ratio [OR] range over all analyses types: 1.33–1.60), with a decreasing OR for increasing distances. In kernel analyses (including all ages), a population-attributable risk between 6.0 and 7.8% was found for a distance of 2000 meters in 2014–2016. The associations were consistent across all years and robust for mutual adjustment for proximity to other animals and for several other sensitivity analyses. However, associations with proximity to poultry farms are not supported by the present study. As the causes of the elevated pneumonia incidence in persons living close to goat farms remain unknown, further research into potential mechanisms is required for adequate prevention.

## Introduction

The evidence regarding the influence of livestock farming on the health of persons living near such farms is mounting [1–5]. Several health effects have been reported, ranging from an increased risk for zoonotic infections like Q fever and methicillin resistant *Staphylococcus aureus* (MRSA) to a lower prevalence of asthma and chronic obstructive pulmonary disease (COPD) [1, 3, 6, 7]. Conversely, persons that have COPD and live close to livestock farms were found to have increased COPD exacerbation [7, 8]. Recently, an increased risk of pneumonia was observed in the Netherlands among residents living close to goat and poultry farms [9–12].

The evidence for this association resulted from several analyses on a large dataset of electronic medical records (2007–2013) of general practitioners’ (GP) patients living in the Dutch provinces Noord-Brabant and Limburg and on a subset of 2,500 of those patients that participated in a medical examination and completed a questionnaire [5, 9–13]. In this area, a major outbreak of Q fever, a zoonosis caused by *Coxiella burnetii*, occurred among goats in 2007–2010 [14] and had a significant public health impact on the nearby human population. Residential proximity to goat farms was associated with an increased incidence of Q fever-related pneumonia [9]. In the years after the epidemic, the association between pneumonia and residence close to goat and other livestock farms was examined in several studies, which mostly indicated that the pneumonia incidence was still elevated among those living near goat farms [11–13]. Potential causes of such elevation remain unclear and it has no clear trace to a single pathogen, as the evidence that microorganisms other than *C*. *burnetii* that can both be found in goats and cause pulmonary complications in humans through indirect transmission is limited to case reports [15].

In two studies investigating cases of pneumonia in relation to proximity to goat farms, a significantly elevated incidence of the disease was also found near poultry farms [9, 12]. A similar association between pneumonia and proximity to poultry was recently reported in Pennsylvania, USA [16], yet in two other studies in the Netherlands, those associations were not significant and positive in only some of the analyses [5, 11]. The associations found in some of the studies were hypothesized to be caused by organic dust emissions from poultry farms, causing a disruption of the upper respiratory tract microbiome [10, 16]. Thus, the elevated incidences of pneumonia were not necessarily caused by zoonotic pathogens. Yet, elevations of pneumonia incidence in residents close to goat and poultry farms may not share that cause, as organic dust emissions from poultry housing are generally much higher than dust emissions from goat farms. In addition, goat farms in the Netherlands generally have natural ventilation and no outdoor access for goats. Such housing may affect potential spread of microorganisms differently than poultry housing, which is generally closed, with mechanical ventilation and, increasingly, with techniques to reduce particulate matter and other pollutants. Exceptions to such closed farms are a minority of free-range farms, where laying hens can freely roam outdoors. Although causal mechanisms remain to be elucidated, the apparent association between pneumonia and proximity to poultry farms has contributed to policy objectives for halving particulate-matter emissions of Dutch poultry farms within 10 years [17]. Furthermore, the apparent association between pneumonia and proximity to goat farms caused most Dutch provinces to temporarily stop issuing building permits for such new and existing farms [18], indicating that a confirmation of results for more recent years is urgent and relevant. Moreover, the data used for previous analyses are from a period partly overlapping the Q-fever epidemic, thus potentially interfering with the associations. Hence, repeating analyses when an outbreak is not occurring is necessary. Therefore, the main aim of this study was to assess the relation between GP diagnoses of pneumonia and several measures of residential proximity to goat and poultry farms in 2014–2016. In addition to focusing on more recent data, we used four types of statistical analyses that were previously used in separate studies to assess the possible influence of these methods on the outcomes.

## Materials and methods

### Study design and study population

A population-based study was performed to analyze the relation between GP diagnoses of pneumonia and living near goat and poultry farms in 2014–2016. The study was conducted in the region where previous research provided evidence of an association between pneumonia and residence close to goat and poultry farms [2, 5, 9, 11, 12, 19, 20]. Using the same registration criteria for participation as previous studies [9], we collected data from 94,295 unique patients registered with 23 GP practices in the area at the time of the study. The selection of these practices, which were all in towns and villages with less than 30,000 inhabitants, varied slightly from earlier studies, because over time, different practices fulfilled quality criteria. Persons living at the same address as a farm location with any livestock (n = 4,081; 4.3%) were excluded from analyses, because these persons were assumed to have an occupational exposure, rather than a residential exposure that is the focus of this study. Persons working on a farm but not living there could not be excluded because occupational data was unavailable, but the percentage of persons excluded was higher than the 1% to 2% of persons working in the agricultural sector in the provinces of Noord-Brabant and Limburg, which comprised the study area. Patients who had multiple home addresses (n = 31) were also excluded. Finally, 90,183 residents were included in this study: 73,510 adults (older than 18 years in 2016) and 16,673 children (18 years or younger in 2016).

### Pneumonia data

The health outcome used in this study was defined as the occurrence of at least one event of GP diagnosed pneumonia during 2014–2016, coded as R81 according to the International Classification of Primary Care (ICPC) [21]. The classification does not allow further specification of R81 to diseases related to pneumonia, like Q fever, with the exception of R81.01 (*Legionella*). In addition, GP diagnoses are generally based on clinical criteria only. Data on pneumonia diagnoses and patients’ age and gender were collected from electronic medical records (EMR). The data provide a complete picture of the registered health status of patients, since in the Netherlands each individual is required to register with one general practice, and GPs then operate as gatekeepers for more specialized health care, usually receiving notification of hospitalization.

### Exposure variables

The proximity of residents to goat farms (n = 95, S1 Table) and poultry farms (n = 881, S1 Table) was quantified in terms of the distance to the nearest farm (m) and the presence or absence of a farm within buffers of 500, 1000, 1500 or 2000 meters from a resident’s home address. Proximity to farms with cattle, pigs, sheep and mink, was also quantified (S1 Table) for use in specific analyses, which are detailed below. Patients’ home addresses were obtained from the EMR data and geocoded with high-resolution Dutch cadastral data from 2015 in which geocodes generally represented coordinates that fell within the building footprint. Farm location and additional information regarding the animal type and numbers were collected from provincial databases on compulsory environmental licenses in 2015 (Bestand Veehouderij Bedrijven of Noord-Brabant and Limburg). Goat and poultry farms, as well as other types of farms, were defined on the basis of definitions described in S1 Table. Exposure variables were calculated using the sf-package in R [22].

In some analyses, a distinction was made between chicken farms and other types of poultry farms. Among chicken farms, a further distinction was made between farms with broilers, where chickens are usually raised in all-in all-out systems in 5 to 10 weeks, and those with laying hens or parent stock (S1 Table), since different farming practices may lead to different pollutant emissions.

### Ethical aspects

The protocol used for the study (number 13/533) has been approved by the Medical Ethical Committee of the University Medical Centre of Utrecht, and the NIVEL Primary Care Database (PCD) complies with the regulations of the Dutch Data Protection Authority and the Dutch law regarding use of health data for epidemiological research purposes (Dutch Civil Law, Article 7:458). According to this law, neither medical ethical approval nor informed consent is required to use EMR data for observational studies on the condition that it contains no directly identifiable data. Medical information and address records were kept separate at all times by use of a Trusted Third Party (Stichting Informatie Voorziening Zorg, Houten).

### Statistical analyses

To evaluate exposure-response relationships between pneumonia and the presence of a specific animal farm within a given distance, we used four different types of statistical analyses that are referred to as single-level and multilevel regression analyses, regression meta-analyses and kernel analyses.

#### Single-level analyses

Single-level logistic regression analyses were performed with the presence of a livestock farm within a buffer of 500, 1000, 1500 or 2000 meters as an exposure proxy, with the glm function in R [23]. The analyses focused on goat and poultry farms, with for poultry a further specification to chicken farms, farms with laying hens or parental stock, broiler farms and farms with other poultry. In addition to these regression analyses with buffers, the effect of the distance to the nearest farm was analyzed by fixed-distance intervals of 500 meters (max 2000 m) in logistic regression analyses. The association between pneumonia and distance to a farm was also visualized in spline plots through generalized additive modeling, with the gam function of the mgcv package in R [24]. Analyses were performed separately for adults and children; all analyses were adjusted for age (linear) and gender and performed for individual years, as well as for the entire period 2014–2016.

#### Multilevel and meta-analyses

Multilevel analyses and meta-analyses were performed to account for potential differences in registration practices between general practitioners. In multilevel regression analyses (glmer function of the lme4 package [25]), GP practice was included as a random intercept and for the remaining part resembled single-level logistic regression analyses. Both multilevel and single-level analyses were performed because although the multilevel analyses may account for differences in the registration of pneumonia between GP practices, they may also lead to over-adjustment of the effect of proximity to livestock farms, as farm types may be clustered close to some GP practices.

Meta-analyses were performed to explore potential differences between GP practices. For each GP practice, logistic regression analyses were performed and the results of these separate analyses were combined in a random effects meta-analysis, via the rma function of the metafor package [26], including only those practices for which there was sufficient exposure; assumed to be indicated by standard errors less than 10.

#### Kernel model

In addition to regression models, a spatial kernel model was used as a complementary approach for analyzing the associations between pneumonia and proximity to livestock farms. A kernel model effectively accounts for the influence of multiple farms because the distance between a home address and all surrounding farms is considered in the analysis. Also, a kernel model provides a relatively simple way to determine a population attributable risk (PAR) [10, 12], which makes results comparable. The model is based on assigning to each type of livestock farm a distance-dependent probability of causing pneumonia in a resident living in the vicinity. This probability is then compared with a uniform background probability through a likelihood ratio test. Further methodological background can be found in Kalkowska et al. [12] and Smit et al. [10].

For the current application, the kernel model was defined on the basis of fixed distances that varied between 500 and 2000 meters with increments of 500 meters. The model was applied to cases of pneumonia in all age groups, without distinguishing between adults and children. In measuring exposure, we delineated six farm types: cattle, goats, mink, pigs, poultry and sheep. For each year, each farm type that gave α < 0.05 in the likelihood ratio test for at least one distance was included in a multivariate kernel model, with independent hazards for proximity to each type of farm. This model provided an indication of the risk increase of living within a certain distance of a livestock farm of a specific type, as well as the PAR.

#### Sensitivity analyses

Three types of sensitivity analyses were performed. First, the effect of changing several assumptions regarding selection criteria and model formulation were tested. In the second type of sensitivity analysis, the effect of leaving out single GP practices from the analyses was assessed. Third, the effect of mutual adjustment for multiple farm types (goats, poultry, pigs, cattle, mink and sheep) in regression analyses was assessed.

## Results

### Study population

In total, 3,610 (4.0%, 3,079 adults, 531 children) residents had at least one GP diagnosed pneumonia episode during 2014–2016 (incidence per 1,000: 15.9 in 2014, 20.2 in 2015, 18.7 in 2016). Among adults, the odds of being diagnosed with pneumonia increased with age (OR 1.05 per year; 95% CI 1.04–1.05; S2 Table), whereas among children this association was reversed (OR 0.90 per year, 95% CI 0.88–0.91; S2 Table). Females were less likely to be diagnosed with pneumonia than males (female adults: OR 0.90, 95% CI 0.84–0.97, female children: OR 0.79, 95% CI 0.66–0.94; S2 Table).

### Single-level analyses

**Fig 1:**
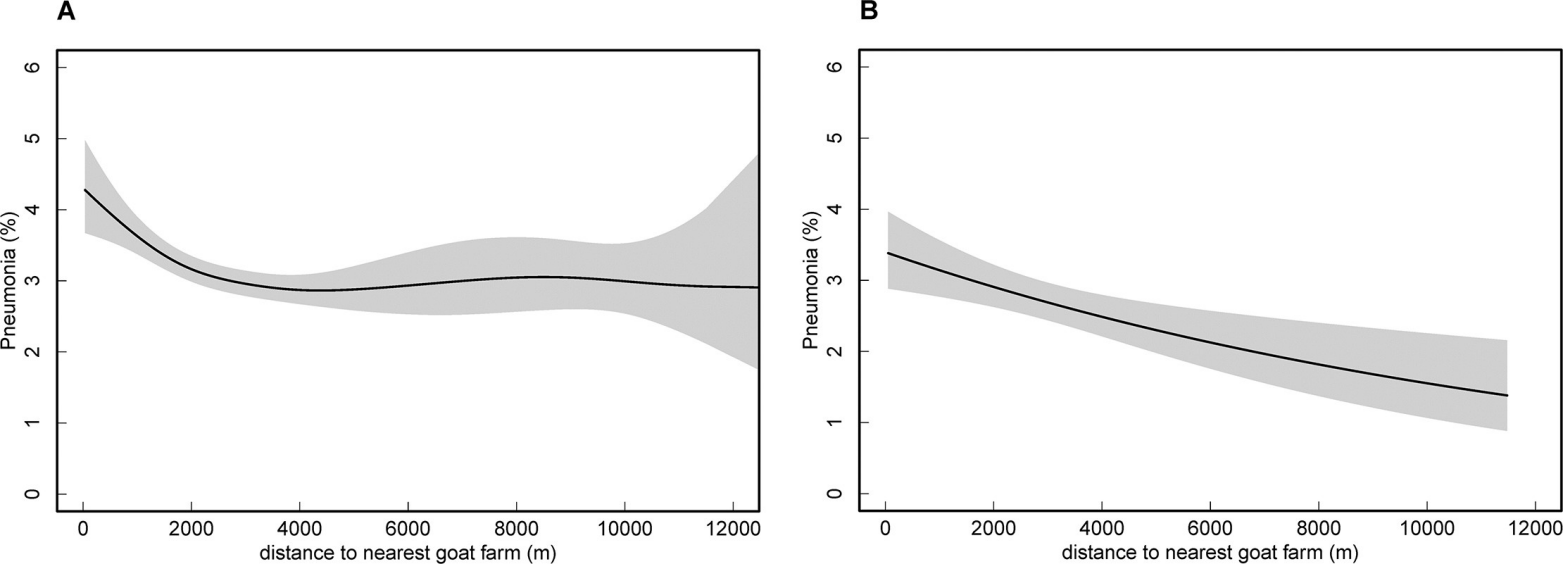
Spline plot for the association between cases of pneumonia and distance to nearest goat farm. The spline is based on generalized additive modelling, with the gam-function (mgcv package R [24]). Panel A shows results for adults (n = 73,510), and panel B shows results for children (n = 16,673). The associations are adjusted for age (linear) and gender; the shaded area is the 95% confidence band. For adults, p (approximate probability that the slope equals 0) < 0.001, for children p = 0.002.

In single-level logistic regression analyses, the association between pneumonia and residence within specified distances to goat farms among adults was statistically significant for all buffers and decreased from 1.60 (95% CI 1.25–2.03) for the presence of a goat farm within 500 meters (reference >500 m) to 1.17 (95% CI 1.09–1.27) for a buffer of 2000 meters (reference >2000 m; Table 1). Pneumonia risk decreased with increasing distance from goat farms until approximately 4 km (Fig 1A, S3 Table). The association between pneumonia and living within a specified distance from any poultry farm was not statistically significant and relatively close to 1 for any buffer in single-level analyses (Table 1); the association did not significantly decrease with distance in spline analyses (not shown). However, odds ratios were higher for proximity to chicken farms and significantly higher than 1 for the presence of broiler farms within buffers of 1500 and 2000 meters (Table 1), with a decreasing pneumonia risk for increasing distances from chicken or broiler farms (S3 Table). Inverse associations with poultry other than chickens were found (Table 1).

**Table 1:** Associations in adults, between the occurrence of a registered pneumonia episode in 2014–2016 and the presence of goat and poultry farms within buffers from the home address (odds ratios, 95% confidence interval).

Buffer	500 meters	1000 meters	1500 meters	2000 meters
Goat farm				
n = 73,510^1^	1.51%	8.47%	19.05%	32.90%
Single-level^2^	1.60 (1.25–2.03)***	1.36 (1.21–1.53)***	1.25 (1.14–1.37)***	1.17 (1.09–1.27)***
Multilevel^3^	1.33 (1.03–1.71)*	1.11 (0.97–1.28).	1.08 (0.97–1.20)	1.07 (0.98–1.18)
Meta-analysis^4^	1.58 (1.10–2.27)*	1.22 (0.97–1.55).	1.08 (0.96–1.22)	1.07 (0.97–1.18)
Poultry farm				
n = 73,510^1^	10.78%	46.76%	79.79%	92.22%
Single-level^2^	1.03 (0.92–1.16)	1.02 (0.95–1.10)	1.00 (0.91–1.09)	0.95 (0.83–1.09)
Multilevel^3^	1.01 (0.89–1.15)	0.98 (0.90–1.07)	1.02 (0.92–1.14)	0.91 (0.77–1.06)
Meta-analysis^4^	1.04 (0.92–1.18)	0.99 (0.89–1.10)	1.00 (0.88–1.14)	0.87 (0.73–1.02).
Chicken farm				
n = 73,510^1^	10.07%	43.13%	74.37%	90.62%
Single-level^2^	1.06 (0.94–1.20)	1.05 (0.97–1.13)	1.06 (0.97–1.15)	1.04 (0.92–1.18)
Multilevel^3^	1.02 (0.90–1.16)	0.98 (0.90–1.07)	1.03 (0.94–1.14)	0.96 (0.82–1.12)
Meta-analysis^4^	1.05 (0.92–1.20)	0.99 (0.89–1.10)	1.02 (0.91–1.15)	0.91 (0.76–1.08)
Farm with laying hens or parent stock				
n = 73,510^1^	8.08%	36.73%	62.29%	83.65%
Single-level^2^	1.08 (0.94–1.23)	1.06 (0.98–1.14)	1.01 (0.94–1.09)	0.95 (0.86–1.05)
Multilevel^3^	1.02 (0.88–1.18)	0.98 (0.90–1.08)	1.02 (0.93–1.11)	0.99 (0.88–1.11)
Meta-analysis^4^	1.08 (0.93–1.25)	1.01 (0.90–1.14)	1.02 (0.93–1.12)	0.97 (0.86–1.10)
Farm with broilers				
n = 73,510^1^	2.51%	12.19%	33.60%	52.55%
Single-level^2^	1.11 (0.87–1.40)	1.11 (0.99–1.24).	1.13 (1.04–1.21)**	1.11 (1.03–1.20)**
Multilevel^3^	1.12 (0.88–1.41)	1.03 (0.92–1.15)	0.98 (0.90–1.07)	0.98 (0.90–1.07)
Meta-analysis^4^	1.23 (0.96–1.56).	1.04 (0.93–1.16)	0.96 (0.86–1.07)	0.96 (0.87–1.06)
Farm with other poultry				
n = 73,510^1^	0.63%	5.27%	14.54%	24.34%
Single-level^2^	0.75 (0.42–1.24)	0.86 (0.71–1.03).	0.82 (0.73–0.92)***	0.90 (0.82–0.98)*
Multilevel^3^	0.88 (0.51–1.51)	0.98 (0.81–1.19)	0.93 (0.82–1.06)	1.10 (0.97–1.23).
Meta-analysis^4^	1.13 (0.66–1.95)	1.05 (0.86–1.28)	0.96 (0.84–1.09)	1.13 (0.99–1.29).

Each odds ratio indicates the outcome of a different regression model.

p<0.15

*p<0.05

**p<0.01

***p<0.001

^1^Percentages indicate the percentage of residents living within a buffer

^2^adjusted for age (linear) and gender

^3^adjusted for age (linear), gender and including GP practice as random intercept

^4^meta-analysis of logistic regression estimates (adjusted for age and gender) for individual GP practices

### Multilevel analyses and meta-analyses

**Fig 2:**
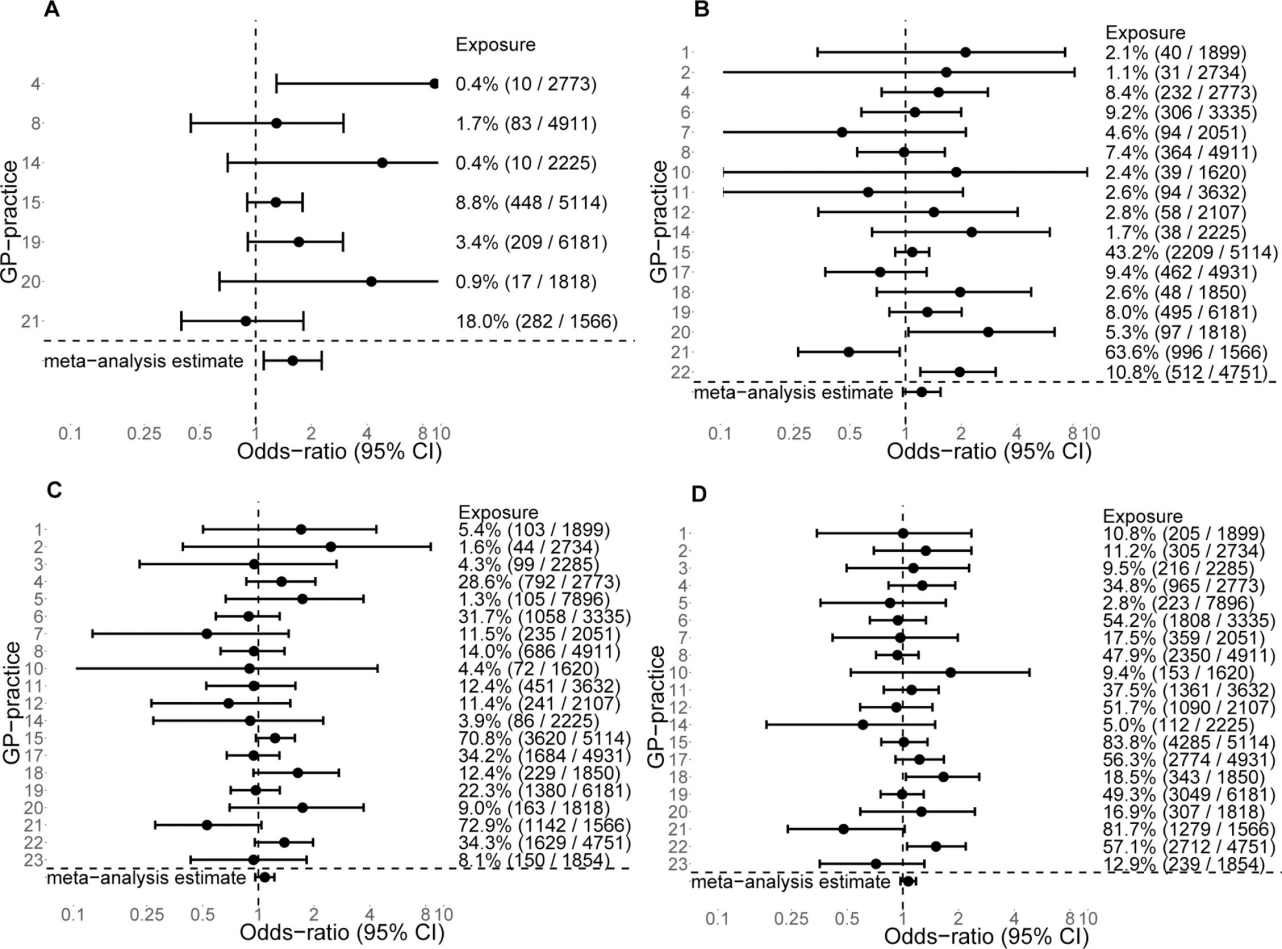
Forest plots for the association between GP-diagnosed pneumonia and the proximity of adults to goat farms. Estimates for each GP practice and meta-analysis estimate are expressed as odds ratios for buffers of 500 meters (panel A), 1000 meters (panel B), 1500 meters (panel C) and 2000 meters (panel D), adjusted for age of the patients (linear) and gender. Estimates for GP practices with no individuals living within a buffer from a goat farm or with standard errors higher than 10 (indicating a low number of residents living within buffer) are not shown. **Logarithmic scale:** confidence bounds are cut off at 0.1 and 10. The meta-analysis estimates are also shown in Table 1.

Accounting for potential differences between GP practices yielded a similar pattern regarding the associations with goat farms among adults in multilevel analyses (OR 500 m: 1.33, 95% CI 1.03–1.71; OR 2000 m: 1.07, 95% CI 0.98–1.18) and in meta-analyses (OR 500 m: 1.58, 95% CI 1.10–2.27; OR 2000 m: 1.07, 95% CI 0.97–1.18), although these analyses showed statistically significant ORs only for buffers of 500 meters (Table 1). Moreover, for this distance, a positive association was found in 6 out of the 7 GP practices (I^2^ = 28.7%, Fig 2). For 1000 meters, a positive association was found for 11 out of 17 GP practices (I^2^ = 44.2%), but for longer distances, less than half (1500 m; I^2^ = 7.9%) or half (2000 m; I^2^ = 1.1%) the practices had a positive association (Fig 2). For analyses regarding the proximity of poultry within 500 meters, all analyses, except those related to poultry other than chickens, showed results similar to those of single-level analyses. However, less consistent results were found for longer distances; significantly elevated ORs in single-level analyses were close to unity in multilevel and meta-analyses (Table 1), while I^2^ ranged from 0 to 25.5%, indicating low heterogeneity.

### Kernel model

Kernel analyses showed positive associations between pneumonia and proximity to goat farms for 2014, 2015 and 2016, with a risk increase ranging from 23.6% to 31.9%, a PAR ranging from 6.0 to 7.2% and a distance of 2000 meters providing the highest likelihood for each year (Table 2). Regarding the association between pneumonia and proximity to poultry farms, only for 2014, a (significant but small) risk increase was found. No significant risk increase was found for 2015 and 2016. This meant that in the multivariate kernel analyses, poultry farms were included in only 2014. Multivariate kernel analyses also included sheep farms for 2014 and 2015 and cattle farms for all years, since significant associations were found in univariate kernel analyses for these farm types (S4 Table).

**Table 2:** Results of multivariate kernel analyses.

Year	2014	2015	2016
Goat farms			
Distance (m)	2000	2000	2000
Risk increase (%)	31.9	23.6	25.4
PAR^1^ (%)	7.8	6.0	7.2
Poultry farms			
Distance (m)	1000	Not applicable	Not applicable
Risk increase (%)	0.6	Not applicable	Not applicable
PAR^1^ (%)	0.4	Not applicable	Not applicable

^1^Population attributable risk

Only the outcomes for poultry and goats are listed; for further details see S4 Table. ‘Distance (m)’ is the distance range for which the best fit in the individual farm-type analysis for the given year was found. The distances used in this multivariate model were the distances that gave the highest likelihood for that specific farm type in that year. ‘Risk increase’ denotes the average increase of the pneumonia risk for individuals living within the distance range of one farm of the given type and is calculated as described in Kalkowska et al. [12].

### Individual years

The association between pneumonia and residence near goat farms was consistent across 2014, 2015 and 2016 (Table 2, S5 Table). For pneumonia among adults living close to a poultry farm, an increased risk was found for 2014 in kernel analyses, but not in other years (Table 2). For 2014, pneumonia in adults was positively associated (p<0.05) with broiler farms as well, for several distances in single level analyses, and for adults living within 500 meters in multilevel and meta-analyses (S5 Table).

### Results for children

ORs for the association between pneumonia among children and proximity to goat farms were similar to those for adults in single-level analyses and meta-analyses, but lower in multilevel analyses (Table 3). Pneumonia risk monotonically decreased with increasing distance (Fig 1B). For proximity to most types of poultry farms, the pattern among children with pneumonia was similar to that among adults; although, in general, associations were weaker than for adults, and negative, rather than positive, associations were found for proximity to broiler farms (Table 3).

**Table 3:** Associations for children, between the occurrence of a registered pneumonia episode in 2014–2016 and the presence of goat and poultry farms within buffers from the home address (odds ratios, 95% confidence interval).

Buffer	500 meters	1000 meters	1500 meters	2000 meters
Goat farm				
n = 16,673^1^	1.59%	9.73%	21.15%	34.80%
Single-level^2^	1.61 (0.85–2.78).	1.29 (0.97–1.67).	1.27 (1.03–1.55)*	1.15 (0.96–1.37).
Multilevel^3^	1.06 (0.58–1.95)	0.94 (0.69–1.29)	1.07 (0.84–1.37)	0.88 (0.70–1.10)
Meta-analysis^4^	1.56 (0.67–3.63)	1.19 (0.83–1.70)	1.27 (0.90–1.79)	0.94 (0.71–1.23)
Poultry farm				
n = 16,673^1^	10.53%	47.86%	80.47%	93.55%
Single-level^2^	0.98 (0.72–1.29)	1.12 (0.94–1.33)	0.90 (0.73–1.11)	1.12 (0.79–1.67)
Multilevel^3^	0.92 (0.68–1.25)	0.98 (0.80–1.20)	0.78 (0.62–1.00)*	0.85 (0.57–1.28)
Meta-analysis^4^	1.10 (0.80–1.51)	0.93 (0.75–1.15)	0.69 (0.53–0.89)**	0.92 (0.86–0.98)**
Chicken farm				
n = 16,673^1^	9.88%	43.63%	74.27%	92.07%
Single-level^2^	0.96 (0.71–1.28)	1.20 (1.00–1.42)*	1.00 (0.82–1.23)	1.19 (0.85–1.71)
Multilevel^3^	0.88 (0.65–1.21)	0.97 (0.80–1.19)	0.77 (0.61–0.96)*	0.83 (0.56–1.23)
Meta-analysis^4^	1.15 (0.82–1.61)	0.91 (0.73–1.13)	0.69 (0.54–0.87)**	0.92 (0.86–0.98)**
Farm with laying hens or parent stock				
n = 16,673^1^	7.72%	37.47%	62.26%	85.14%
Single-level^2^	1.07 (0.77–1.46)	1.23 (1.03–1.47)*	1.18 (0.98–1.41).	1.26 (0.97–1.66).
Multilevel^3^	0.92 (0.65–1.29)	1.00 (0.81–1.23)	0.99 (0.80–1.22)	1.13 (0.83–1.52)
Meta-analysis^4^	1.34 (0.87–2.06)	0.96 (0.77–1.20)	0.93 (0.74–1.17)	0.83 (0.48–1.43)
Farm with broilers				
n = 16,673^1^	2.64%	12.54%	34.35%	54.63%
Single-level^2^	0.60 (0.28–1.09).	0.92 (0.70–1.19)	0.85 (0.70–1.02).	1.01 (0.85–1.20)
Multilevel^3^	0.69 (0.35–1.36)	0.85 (0.64–1.12)	0.67 (0.54–0.82)***	0.77 (0.63–0.96)*
Meta-analysis^4^	1.65 (0.79–3.44)	0.96 (0.71–1.29)	0.67 (0.54–0.84)***	0.72 (0.57–0.90)**
Farm with other poultry				
n = 16,673^1^	0.49%	5.28%	13.36%	22.77%
Single-level^2^	1.05 (0.26–2.84)	0.81 (0.52–1.21)	0.70 (0.52–0.92)*	0.59 (0.46–0.75)***
Multilevel^3^	1.41 (0.44–4.58)	1.25 (0.79–1.96)	1.07 (0.77–1.51)	0.85 (0.62–1.17)
Meta-analysis^4^	3.33 (0.92–12.02).	1.47 (0.89–2.45).	1.33 (0.79–2.25)	1.23 (0.65–2.32)

Each odds ratio indicates the outcome of a different regression model.

p<0.15

*p<0.05

**p<0.01

***p<0.001

^1^Percentages indicate the percentage of residents living within a buffer

^2^adjusted for age (linear) and gender

^3^adjusted for age (linear), gender and including GP practice as random intercept

^4^meta-analysis of logistic regression estimates (adjusted for age and gender) for individual GP practices

### Sensitivity analyses

In the first type of sensitivity analyses, change of assumptions regarding the selection of persons and model specification generally led to a change in ORs for proximity to goat and poultry farms of less than 10% (S6 and S7 Tables). Specification of a model with the number of animals in a buffer as an exposure variable, rather than presence or absence of a farm type, gave similar patterns for both goats and poultry (data not shown). In the second type of sensitivity analyses, in which single GP practices were excluded from the analyses, ORs related to proximity to goat farms could be affected by more than 25% in single-level analyses and meta-analyses, but were less affected in multilevel analyses (S8 Table); the direction of the association was not affected. In analyses in which the proximity to different types of animals were mutually adjusted for each other, the effect sizes were slightly affected, but results still showed clear positive associations for proximity to goat farms and no associations for proximity to poultry farms (S9 Table). In these and univariate analyses, significantly positive and negative associations between pneumonia and the proximity to farms other than goat or poultry farms were found (S3, S5 and S9 Tables). A positive association was shown with proximity to sheep farms, albeit less strong when compared to associations with proximity to goat farms. For cattle, pigs and mink, the positive and negative associations were generally less consistent over time between children and adults or between types of analyses, compared to associations with proximity to goat farms (S3, S5 and S9 Tables).

## Discussion

In this study, we found a consistent association between proximity to goat farms and pneumonia, with an estimated PAR between 6.0 and 7.8% during 2014–2016. This estimate corresponded to about 1.2 to 1.3 pneumonia cases per 1000 residents in the study population that were attributable to living in the vicinity of goat farms, with the total incidence between 15.9 and 20.2 cases. Results thus confirm observations during 2009–2013 [9, 11–13]. Multilevel regression analyses and regression meta-analyses generally showed patterns similar to single-level regression analyses, but odds ratios were closer to unity (Table 1). This smaller effect size may be explained by the lower exposure contrast associated with the random-effect term in the multilevel analyses and with the stratification of the data by GP practice for the meta-analyses. The significantly positive association in the multilevel analyses and the association with several independent GP practices, which resulted in limited heterogeneity, strongly indicate that outcomes in single-level analyses were not driven by differences in registration practices between general practitioners. Furthermore, results were only slightly sensitive to adjustment for proximity to other types of animals and several alternative assumptions.

The indications for an association between pneumonia and proximity to poultry farms are weak, with no significant associations in most analyses. In previous studies in the Netherlands that found an association, the increased risk of pneumonia due to proximity to poultry farms was generally lower than that due to proximity to goat farms [9, 12]. In the current study, the size of the association with poultry farms was even smaller, with only a small but significant association among all age groups in kernel analyses in 2014. Also in 2014, associations with broiler farms were significantly positive in regression analyses among adults, but not significant and in most analyses negative among children. The results may thus reflect disappearance of an effect, but they may also indicate a weak effect or a chance finding, as associations in two other studies in the Netherlands were not significant and positive in only some of the analyses [5, 11]. Methodological differences are less likely to explain the variations in results, because previous kernel analyses differ from those in this study only in the use of older data on health outcomes and farm locations [12]. Nevertheless, a study in Pennsylvania, USA, where farming practices and rural conditions may differ from those in the Netherlands, found an association between poultry-farm proximity and community-acquired pneumonia [16].

Results for children generally showed similar patterns compared to those for adults. Yet, contrasting results were found for some analyses, particularly regarding broilers, which are difficult to explain. Moreover, ORs for children generally had wider CIs and limited statistical significance for shorter distances than those for adults, likely due to the smaller study population. Despite similar patterns, the spline plots suggest that the risk of pneumonia for children is increased for longer distances from goat farms than the risk for adults, but such a difference is not directly supported by results from analyses on distance intervals (S3 Table).

Single-level analyses, multilevel analyses and meta-analyses were used to account for the trade-off between bias related to potential differences in reporting between GPs and the limited exposure contrast among patients registered with the same GP. This trade-off cannot be resolved by any individual analysis for this study, but the combination of analyses indicate that results were not driven by differences in reporting between GPs or a limited exposure contrast. The resemblance of results for single-level and multilevel analyses regarding an association with goat farms was not observed in previous studies regarding 2007–2013. Over this period, in one study a multilevel model was used to analyze the association between pneumonia and the presence of a goat farm within 500 meters [5]. Results of this study did not show the positive association found in single-level analyses for the same exposure variable [9, 11, 13], yet the studies differed in several other aspects besides the multilevel structure.

Although this study suggests that residents living near goat farms may be at increased risk for pneumonia, the causes of this association remain unknown. Pneumonia is the main presentation of human Q fever. It is unlikely that Q fever is the cause of the currently found association because goats are vaccinated against *C*. *burnetii*, and, since 2013, the reported incidence of Q fever has been the same as before the epidemic of 2007–2010 (with fewer than 30 notified cases per year compared to several thousand during 2009, the peak year of the outbreak) [27]. For associations with poultry farms, a relationship to particulate matter emissions from these farms has been hypothesized [10, 16]. However, this hypothesis is unlikely to explain the association with goat farms since these emissions from goat farms are generally much lower than those from poultry farms.

The use of EMR data for this study can be considered a strength because it provided a relatively large study population with an accurate epidemiological denominator but also has some limitations. One limitation of EMR is that such data do not provide information on possible confounders such as smoking and socio-economic status, so no adjustment was possible. Data on other diseases that may be a risk factor for pneumonia, like chronic obstructive pulmonary disorder (COPD), are available in EMR data, but these were not included because possible interactions with such health outcomes were out of the scope of this study; such health outcomes have been studied separately [5, 28, 29]. Previous research on a smaller population that adjusted for these potential confounders and comorbidities found limited influence on the associations between pneumonia and proximity to goat farms [11]. Another potential confounding factor is occupational exposure, although we aimed to reduce that exposure as much as possible by excluding persons assumed to be living on livestock farms. Another factor is the season in which pneumonia was diagnosed, given that atypical microorganisms causing pneumonia may be detected more often outside the winter months (weeks 20–39), whereas during winter (weeks 40–19) typical pathogens are more common [30]. EMR data also do not provide information on a potential etiologic agent, since GPs generally base their diagnoses on clinical criteria only, and led it follow by presumptive treatment with antibiotics, which is in line with prevailing national and international professional guidelines, [31, 32]. The lack of information on a potential etiologic agent, which is usually unavailable in self-reported data and in most of the hospital data, makes inferences about the nature of associations harder.

Another limitation of the study is the limited precision of exposure assessment, because it relies on a proxy of distances between homes and farms, with no information on duration and mode of exposure. This limited precision makes inference of potentially small associations harder. Rather than home location, the proximity of the workplace or school to farms could also indicate exposure, as persons generally spend a large part of their day at such locations. Unfortunately, no information on locations or occupation was available. Nevertheless, a previous study specifically studying mobility close to goat farms, found that such mobility only marginally contributed to pneumonia risk compared to the significantly elevated risk of living near goat farms, which suggests that residential proximity may be an appropriate measure for exposure [13]. Apart from taking into account the location of persons during their day, exposure assessment could also be improved by considering factors like wind direction [33, 34], which will be subject to future research. Finally, another weakness regarding exposure assessment is the precision of farm location data; although these data are used for environmental licensing, they may not be fully up to date or contain exact coordinates for all farm locations. Yet, distances to farm locations in this dataset have been used as suitable predictors for measured air pollutants [33, 34], and potential imprecision should not lead to differential exposure misclassification.

In conclusion, the results of this study suggest that residents within proximity of goat farms have a higher risk for pneumonia of unknown etiology. Further research with molecular microbial techniques and more accurate diagnostic information would help in the understanding of the increased risk of pneumonia near goat farms and the determination of adequate preventive measures. Such research should primarily focus on local residents but because the exposure of goat farmers is generally higher than that of local residents, studies specifically targeting the farmers may provide more information regarding potential etiological agents as well. The present study is less conclusive about an association between pneumonia and the proximity to poultry farms.

## Supporting information

S1 Table Cut-off points for the minimum number of animals per farm as used for regression analyses, number of farms in the study area (postal codes starting with 52–60) and mean number of animals per farm. (XLSX)

S2 Table Study population characteristics. (XLSX)

S3 Table Donut analyses for adults: associations between having had a registered pneumonia-episode in 2014–2016 and the presence of farms within distance intervals (donuts) from an adult's home address (odds ratios, 95% confidence interval). (XLSX)

S4 Table Detailed results of kernel analyses. Results for individual and multivariate kernel analyses for community-acquired pneumonia around different farm types by year.
(XLSX)

S5 Table Buffer analyses per year for adults: associations between having had a registered pneumonia episode in 2014, 2015 and 2016 and the presence of farms within buffers from an adult's home address (odds ratios, 95% confidence interval). (XLSX)

S6 Table Sensitivity to assumptions among adults. (XLSX)

S7 Table Sensitivity to assumptions among children. (XLSX)

S8 Table Leave-one-out analyses for analyses of associations between pneumonia in 2014–2016 and living within 500 meters from a goat farm (odds ratios, 95% confidence interval). (XLSX)

S9 Table Mutual adjustment for 6 animal types among adults: associations between having had a registered pneumonia episode in 2014–2016 and the presence of farms within buffers from an adult's home address. (XLSX)
